# Associations between dietary intake and glucose tolerance in clinical and metabolomics-based metabotypes

**DOI:** 10.1186/s12263-023-00721-6

**Published:** 2023-03-10

**Authors:** Amanda Rundblad, Jacob J. Christensen, Kristin S. Hustad, Nasser E. Bastani, Inger Ottestad, Kirsten B. Holven, Stine M. Ulven

**Affiliations:** 1 Department of Nutrition, Institute of Basic Medical Sciences, University of Oslo, P.O. Box 1046 Blindern, 0317 Oslo, Norway; 2 National Advisory Unit on Familial Hypercholesterolemia, Department of Endocrinology, Morbid Obesity and Preventive Medicine, Oslo University Hospital, Oslo, Norway

**Keywords:** Metabotyping, Metabotypes, Glucose tolerance, Diet

## Abstract

**Background:**

Metabotyping is a novel concept to group metabolically similar individuals. Different metabotypes may respond differently to dietary interventions; hence, metabotyping may become an important future tool in precision nutrition strategies. However, it is not known if metabotyping based on comprehensive omic data provides more useful identification of metabotypes compared to metabotyping based on only a few clinically relevant metabolites.

**Aim:**

This study aimed to investigate if associations between habitual dietary intake and glucose tolerance depend on metabotypes identified from standard clinical variables or comprehensive nuclear magnetic resonance (NMR) metabolomics.

**Methods:**

We used cross-sectional data from participants recruited through advertisements aimed at people at risk of type 2 diabetes mellitus (*n* = 203). Glucose tolerance was assessed with a 2-h oral glucose tolerance test (OGTT), and habitual dietary intake was recorded with a food frequency questionnaire. Lipoprotein subclasses and various metabolites were quantified with NMR spectroscopy, and plasma carotenoids were quantified using high-performance liquid chromatography. We divided participants into favorable and unfavorable clinical metabotypes based on established cutoffs for HbA1c and fasting and 2-h OGTT glucose. Favorable and unfavorable NMR metabotypes were created using k-means clustering of NMR metabolites.

**Results:**

While the clinical metabotypes were separated by glycemic variables, the NMR metabotypes were mainly separated by variables related to lipoproteins. A high intake of vegetables was associated with a better glucose tolerance in the unfavorable, but not the favorable clinical metabotype (interaction, *p* = 0.01). This interaction was confirmed using plasma concentrations of lutein and zeaxanthin, objective biomarkers of vegetable intake. Although non-significantly, the association between glucose tolerance and fiber intake depended on the clinical metabotypes, while the association between glucose tolerance and intake of saturated fatty acids and dietary fat sources depended on the NMR metabotypes.

**Conclusion:**

Metabotyping may be a useful tool to tailor dietary interventions that will benefit specific groups of individuals. The variables that are used to create metabotypes will affect the association between dietary intake and disease risk.

**Supplementary Information:**

The online version contains supplementary material available at 10.1186/s12263-023-00721-6.

## Background

Type 2 diabetes mellitus (T2DM) is one of the major causes of death globally, and the number of people with T2DM increases rapidly [1]. The important risk factors for T2DM include obesity, an unhealthy diet, and a sedentary lifestyle [2]. Evidence indicates that it is possible to prevent T2DM and improve glycemic control by replacing saturated with polyunsaturated fats and refined grain with whole grain, having a moderate alcohol consumption, limiting intake of processed meat and sugar-sweetened beverages, as well as consuming nuts, coffee, and low-fat dairy [3–5]. However, studies are inconsistent, in part because metabolic characteristics may influence diet-disease associations [6, 7].

The metabolome is the totality of small molecules present in cells, tissues, or body fluids. The genome, transcriptome, and proteome as well as the gut microbiota and environmental factors, such as diet and drugs, produce the metabolome [8]. Hence, the interactions that shape the metabolome also shape disease risk [9]. A metabolic phenotype, also called metabotype, refers to a group of individuals with a similar metabolic profile [10]. Metabotyping can be used in personalized medicine to predict drug response, and investigating associations between metabotypes and disease risk may provide insight into risk factors and improved treatment strategies [11, 12]. Metabotypes can be generated using different approaches, using a few selected or a large variety of metabolites, in a fasting state or as a response to an intervention [6]. In the simplest sense, metabotyping can be based on diagnosis criteria or subgrouping of patients, while a more complex approach is to metabotype based on omics-technologies, including metabolomics, transcriptomics, and epigenomics [6]. However, to justify the use of expensive and time-consuming technologies to generate metabotypes, these omics-based metabotypes should be more useful than the more simple clinical metabotypes.

The metabolic phenotype may modify the response to dietary intake on risk of lifestyle diseases [6, 13]. Hence, metabotyping can be used to identify and stratify groups of individuals that respond differently to dietary intake that therefore could benefit from targeted nutritional recommendations [14]. Previous randomized controlled trials that did not succeed to improve glucose tolerance by dietary interventions may have provided dietary interventions that are not optimal for the whole group [15, 16]. Hence, in future studies, the use of metabotyping may guide researchers to tailor dietary interventions to metabotypes that are more likely to benefit from specific dietary modifications.

With this in mind, we aimed to investigate the association between long-term habitual dietary intake and glucose tolerance in metabotyped subjects, based on standard clinical variables or comprehensive NMR metabolomics. We hypothesized that metabotypes with more unfavorable characteristics would show stronger associations between glucose tolerance and dietary intake than the more favorable metabotypes.

## Subjects and methods

### Participants

This cross-sectional study was conducted between August 2018 and September 2019 at the University of Oslo, Norway. Participants were recruited through advertisements, aimed to reach those at risk of T2DM, in social media and medical practices at the University of Oslo. After a telephone interview, individuals not diagnosed with T2DM or using drugs affecting blood glucose levels attended a screening visit to screen for eligibility to participate in a randomized controlled trial examining the effects of intake of salmon fish protein [17]. Data collected at this screening visit was used in the current cross-sectional sub study. The study was conducted according to the guidelines laid down in the Declaration of Helsinki. All participants gave their written informed consent, and the Regional Ethics Committee for Medical Research in South-East Norway approved the study. The study was registered at ClinicalTrials.gov (ClinicalTrials.gov Identifier: NCT03764423).

### Clinical assessment

The participants’ body weights were measured on a digital scale with a stadiometer (SECA GmbH, Germany) with light clothing and without shoes, and waist circumference was measured according to WHO guidelines [18]. Blood pressure was measured in the non-dominant arm after a 10-min rest by a Carescape V100 monitor (GE Healthcare, USA). Three measurements were obtained with a 1-min interval, and the average of the last two measurements was calculated. Information about the use of hormonal contraceptives and other drugs, as well as information about the menopausal status was obtained by a questionnaire.

### Blood sampling, OGTT, and laboratory analyses

The participants were instructed to avoid consuming alcohol and doing strenuous physical activity the day before the visit. After an overnight fast, venous blood samples were drawn. For the oral glucose tolerance test (OGTT), the participants were instructed to drink a 75-g anhydrated glucose drink (Esteriplas, Portugal) in less than 5 min within 10 min after a fasting venous blood sample. Then, the participants were instructed to avoid eating, drinking, and doing any activity and to remain in the waiting room until the postprandial blood samples were drawn 2 h after finishing the glucose drink.

We obtained serum from silica gel tubes (Becton, Dickinson and Company) kept at room temperature for 30–60 min before centrifugation (1500 g, 15 min). Plasma was obtained from EDTA tubes (Becton, Dickinson and Company) that were immediately placed on ice and centrifuged within 10 min (2000 g, 4 °C, 15 min). Serum concentrations of standard biochemical parameters, including fasting and 2-h OGTT glucose, insulin, HbA1c, triglycerides, total-, LDL-, and HDL-cholesterol and high-sensitive C-reactive protein were measured by standard methods at an accredited routine laboratory (Fürst Medical Laboratory, Norway).

### NMR spectroscopy

About 250 metabolic biomarkers were quantified from EDTA plasma using a commercial high-throughput nuclear magnetic resonance (NMR) spectroscopy platform (Nightingale Health, www.nightingalehealth.com). This platform quantifies metabolites in three molecular windows; lipids, lipoproteins, and low molecular weight metabolites. The lipids quantified include SFA, MUFA, and PUFA, as well as some specific fatty acids, sphingomyelins, and cholines. Among lipoproteins, 14 lipoprotein subclass particles are quantified, as well as the particles’ concentrations of total lipids, phospholipids, total and free cholesterol, cholesteryl esters, and triglycerides. The lipoprotein subclasses are defined by their average diameter; > 75 nm, 64 nm, 53.6 nm, 44.5 nm, 36.8 nm, and 31.3 nm for the six VLDL subclasses; 28.6 nm for intermediate density lipoprotein (IDL); 25.5 nm, 23.0 nm, and 18.7 nm for the three LDL subclasses; and 14.3 nm, 12.1 nm, 10.9 nm, and 8.7 nm for the four HDL subclasses. The low molecular weight metabolites quantified include amino acids, albumin, creatinine, glycoprotein acyls, ketone bodies, and glycolysis-related metabolites. In this study, variables expressing ratios and percentages were removed, and a total of 168 metabolites were used for clustering analyses (Supplemental file 1). Details of this NMR metabolomics platform have previously been described [19, 20].

### Dietary assessment

We assessed habitual food intake from the preceding year using food-frequency questionnaires (FFQ) [21]. The FFQ included questions about the frequency of intake and portion sizes for 270 food items. From the FFQ, we obtained data on intake of food items (g/person/day) and as intake of nutrients as energy percent (E%). Food groups were constructed manually by categorizing food items as shown in Table 1.

**Table 1:** Grouping of food items into food groups used in regression analyses

Food group	Food items
Vegetables	Carrot, rutabaga, cabbage, cauliflower, broccoli, onion, lettuce, cucumber, squash, tomato, bell pepper, spinach, peas, beans, mushroom, canned vegetables, pickled vegetables, vegetable dishes, and vegetarian products
Nuts and seeds	Salted and unsalted nuts, seeds
Rice	Rice
Pasta	Pasta and pasta dishes
Potato	Boiled, pan fried, fried, and mashed potatoes, potato gratin, potato salad
Whole grain	Whole grain (> 50%) bread, crisp bread, grains, oatmeal, unsweetened cereals, porridge
Refined grain	White bread, bread with < 50% whole grain flour, sweetened cereals, tortillas, buns, cookies, cakes
Fruits	Citrus fruit, apple, pear, other fresh fruits and berries, fruit dishes and products, dried fruits
Poultry	Poultry and poultry sausages
Red meat	Beef, game, sheep
Processed meat	Salted, cured and canned meat, minced meat, sausages, ham, liver pâté, other meat products
Total fish and shellfish	Cod, pollock, salmon, trout, herring, mackerel, shellfish, sushi, fish spreads, fish products, breaded fish, other fish
Lean fish	Cod, pollock, breaded fish, fish products
Fatty fish	Salmon, trout, herring, mackerel
Total dairy	Milk, yoghurt, flavored milk, fermented milk, quark, skyr, cream and sour cream, milk and cream products, ice cream, cheese, low-fat cheese, whey cheese, butter
Low-fat dairy	Milk, yoghurt, flavored milk, fermented milk, quark, skyr, low-fat cheese (< 20% fat), low fat whey cheese
High-fat dairy	Cream, sour cream, ice cream, Cheese (> 20% fat), whey cheese, butter
Fermented dairy	Yoghurt, fermented milk, quark, skyr, cheese
Non-fermented dairy	Milk, flavored milk, ice cream, milk and cream products, whey cheese, butter
Oil and oil products	Vegetable oils, mayonnaise, salad dressings, mayonnaise based salads
Margarine	Margarine and low-fat margarine
Coffee	Coffee
Tea	Tea
ASB	Artificially sweetened soda, lemonade and energy drinks
Alcoholic beverages	Beer, wine, spirits
Sweets	Jam, marmalade, juice, sugar, honey, syrup, sweet bread spreads, chocolate, candy, desserts, popsicle, SSB, potato chips, other snacks
Egg	Egg, omelet, scrambled eggs

*ASB* artificially sweetened beverages, *SSB* sugar-sweetened beverages

### Quantification of plasma carotenoids

The EDTA plasma concentration of the carotenoids lutein, zeaxanthin, β-cryptoxanthin, α-carotene, β-carotene, and lycopene were determined by high-performance liquid chromatography with ultraviolet detection (HPLC–UV) as described previously [22]. Briefly, plasma samples were precipitated by the addition of a 4.5 times volume of isopropanol containing internal standard. Plasma calibrators and controls were quantified against the standardized reference material 968c from the National Institute of Standards and Technology.

### Generation of metabotypes and statistical analyses

Because we wanted to address how the large heterogeneity in metabotype generation may determine the findings in metabotype studies, we generated metabotypes in two distinct ways. The clinical metabotypes were generated by categorizing participants based on thresholds of a small set of clinically relevant variables. The NMR metabotypes, on the other hand, were generated by clustering participants based on comprehensive omics data.

#### Clinical metabotypes

The participants with either fasting glucose > 5.5 mmol/L, 2-h glucose > 6.4 mmol/L or HbA1c ≥ 5.8% were categorized as *unfavorable* clinical metabotype. All other participants were categorized as *favorable* clinical metabotype.

#### Clustering of participants into NMR-based metabotypes

After imputing missing data using the k-nearest neighbor and scaling to mean = 0 and SD = 1, we used NMR metabolomics data to cluster participants into metabotypes by three different approaches. In the first approach, we clustered the scaled data directly, using k-means clustering. In the second approach, we did principle component analysis (PCA) with the scaled NMR data as input and clustered the participants using the first four principal components using k-means clustering. Finally, in the third approach, we regressed all the scaled NMR variables on sex, age, BMI, smoking, and use of statins with a linear model, before we clustered the participants using the residuals from the regression model.

#### Associations between a 2-h glucose and food intake, and interactions with metabotypes

The intake of most food groups were right skewed; hence, all food group variables were log-transformed (log(x + 1)) to obtain more normally distributed variables. We used linear models to analyze if there were associations between 2-h glucose and intake of food groups, adjusted for sex, age, BMI, smoking, statin use, and energy intake (kJ) in the whole sample. To analyze if there were food group-metabotype interactions on the association with a 2-h glucose, we used linear models with a food group-metabotype interaction term, adjusted for the same covariates. These interaction analyses compare the favorable to the unfavorable clinical metabotype and the favorable to the unfavorable NMR metabotype. There are no statistical comparisons between metabotyping strategies. The corresponding models were used for carotenoid-metabotype interaction analyses. To visualize the associations between intake of food groups and 2-h glucose, we regressed 2-h glucose on the food groups, adjusted for the same covariates, but without the interaction term, with data from each metabotype separately. The corresponding analyses for nutrient intake as E% were performed in the same manner, except that these analyses were not adjusted for energy intake. Finally, we investigated the important confounding factors age and sex using a linear model with a 2-h glucose as the dependent variable and with an interaction term between the food variables and these factors. All statistical analyses were performed in R, version 4.0.3 [23].

## Results

### Characteristics of the metabotypes

Clinical, NMR, and FFQ data, after excluding participants with an energy intake > 20 000 kJ (*n* = 4), were available for 203 participants that were used for analyses in this study. No participants were excluded for having an energy intake < 4 000 kJ. The favorable clinical metabotype (*n* = 99) had lower fasting and 2-h glucose and HbA1c than the unfavorable clinical metabotype (*n* = 104, Table 2), as expected, as these were the variables we used to generate the clinical metabotypes. In addition, the favorable clinical metabotype was younger and had more premenopausal women and women using hormonal contraceptives, a lower BMI, waist circumference, insulin, and CRP and higher Lp(a) than the unfavorable clinical metabotype.

**Table 2:** Characteristics of the clinical metabotypes

	Favorable clinical metabotype (*n* = 99)*	Unfavorable clinical metabotype (*n* = 104)**
*n* (%)		
Men	31 (31.3)	39 (37.5)
Statin users	5 (5.1)	24 (23.1)
Anti-inflammatory drug users	3 (3)	4 (3.8)
Premenopausal women	48 (71)	18 (28)
Women using hormonal contraceptives	27 (40)	5 (8)
Mean (SD)		
Age (years)	44 (12)	55 (10)
BMI (kg/m^2^)	30.9 (5.1)	33.3 (4.7)
Waist circumference (cm)	101.2 (14.0)	111.9 (12.0)
HbA1c (mmol/mol)	27 (14)	33 (16)
Total-C (mmol/L)	5.0 (0.9)	5.1 (1.0)
LDL-C (mmol/L)	3.3 (0.8)	3.5 (1.0)
Median (IQR)		
HDL-C (mmol/L)	1.4 (0.5)	1.3 (0.4)
Triglycerides (mmol/L)	1.15 (0.56)	1.46 (1.02)
Lp(a) (mg/L)	316 (498)	202 (425)
Fasting glucose (mmol/L)	4.9 (0.4)	5.8 (0.8)
Insulin (pmol/L)	61 (42)	100 (77)
2-h glucose (mmol/L)	4.7 (1.4)	6.6 (2.6)
Systolic BP (mmHg)	116 (14)	124 (21)
Diastolic BP (mmHg)	68 (12)	72 (13)
CRP (mg/L)	2.1 (3.1)	3.8 (5.1)

*BMI*, body mass index; *HbA1c*, glycated haemoglobin; *C*, cholesterol; *LDL*, low-density lipoprotein; *HDL*, hgh-density lipoprotein; *Lp(a)*, lipoprotein a; *BP*, blood pressure; *CRP*, C-reactive protein

^*^Age, *n* = 96; BMI and waist circumference, *n* = 98; Lp(a), *n* = 50; 2-h glucose, *n* = 97

^**^Age and BMI, *n* = 103; fat mass, *n* = 98; LDL-C and HDL-C, *n* = 102; Lp(a), *n* = 66

**Fig. 1:**
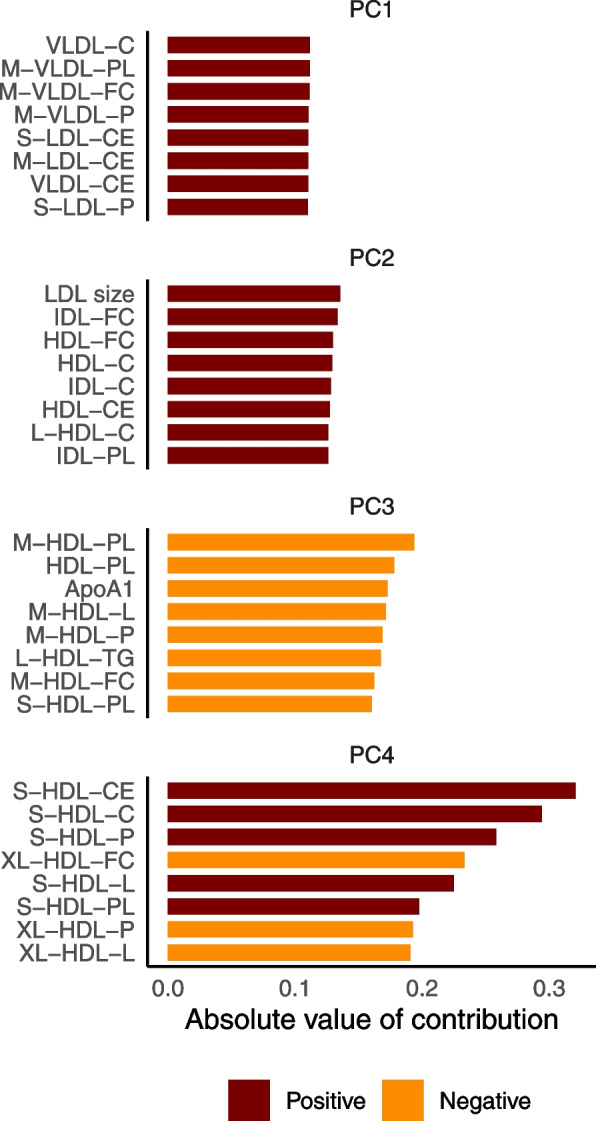
The variables with the largest contribution to the separation of the first four principal components. ApoA1, apolipoprotein A1, C, cholesterol, CE, cholesteryl esters, FC, free cholesterol, HDL, high-density lipoprotein, IDL, intermediate-density lipoprotein, L, large, LDL, low-density lipoprotein, M, medium, P, particle concentration, PC, principal component, PL, phospholipids, S, small, VLDL, very-low-density lipoprotein, XL, extra-large

**Fig. 2:**
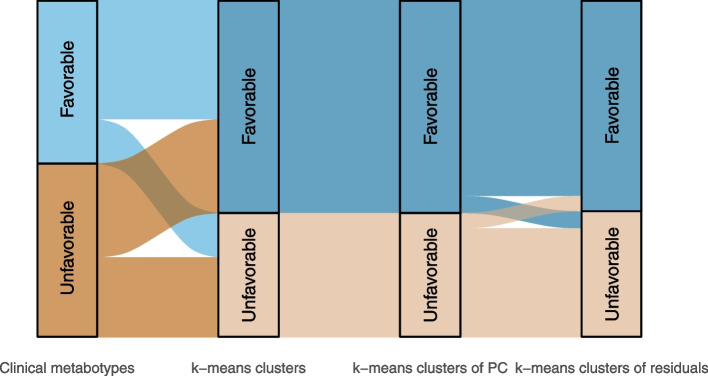
Separation of participants into the favorable and unfavorable clinical and NMR metabotypes. The NMR metabotypes were generated using three approaches: k-means clusters based on NMR metabolomics data, k-means clusters of the four first principal components, and k-means clusters of residuals from regression analyses adjusted for age, sex, body mass index, statin use, and smoking. PC, principal components

We generated the NMR metabolomics-based metabotypes using three different approaches. Firstly, the participants were clustered into two metabotypes based on scaled NMR metabolomics data directly. Secondly, we did a PCA on NMR metabolomics data. The first four principal components (PC) explained about 85% of the variation (Supplemental Fig. 1), and we used these four PCs to cluster the participants into two metabotypes. The concentration of VLDL, LDL, IDL, and HDL particles and lipids, including cholesterol, were important contributors for the first four PCs (Fig. 1). Finally, we wanted to capture variation in the NMR metabolomics data independently of variation in sex, age, BMI, statin use, and smoking status. Hence, residuals from regression models with these variables as independent variables were clustered into two metabotypes. The separation of participants into the clinical metabotypes and the NMR metabotypes based on the three different clustering approaches are shown in Fig. 2, and the participant clusters are shown in Supplemental Fig. 2. As the different clustering approaches resulted in very similar metabotypes, we continued our analyses with the clusters from direct clustering of NMR data and from clustering of the first four PCs. The NMR-based metabotypes are hereafter called *favorable* NMR metabotype and *unfavorable* NMR metabotype.

The favorable NMR metabotype (*n* = 127) had lower total- and LDL-C and triglycerides and higher HDL-C than the unfavorable NMR metabotype (*n* = 76, Table 3). In addition, there were more men in the unfavorable than the favorable metabotype. The age, proportion of premenopausal women and women using hormonal contraceptives, BMI, waist circumference, and glucose-related variables were more similar between the NMR metabotypes than between the clinical metabotypes.

**Table 3:** Characteristics of the NMR metabotypes

	Favorable NMR metabotype (*n* = 127)*	Unfavorable NMR metabotype (*n* = 76)**
*n* (%)		
Men	30 (23.6)	40 (52.6)
Statin users	22 (17.3)	7 (9.2)
Anti-inflammatory drug users	7 (5.5)	0 (0)
Premenopausal women	54 (56)	12 (33)
Women using hormonal contraceptives	29 (30)	3 (8)
Mean (SD)		
Age (years)	49 (13)	51 (11)
BMI (kg/m^2^)	31.8 (5.1)	32.7 (4.9)
Waist circumference (cm)	105.0 (14.5)	109.6 (12.8)
HbA1c (mmol/mol)	29 (15)	32 (16)
Total-C (mmol/L)	4.6 (0.8)	5.7 (0.9)
LDL-C (mmol/L)	3.0 (0.7)	4.1 (0.9)
Median (IQR)		
HDL-C (mmol/L)	1.4 (0.5)	1.1 (0.3)
Triglycerides (mmol/L)	1.06 (0.45)	1.9 (0.99)
Lp(a) (mg/L)	309 (531)	227 (341)
Fasting glucose (mmol/L)	5.1 (0.9)	5.5 (0.9)
Insulin (pmol/L)	68 (59)	96 (71)
2-h glucose (mmol/L)	5.1 (2.2)	5.8 (2.5)
Systolic BP (mmHg)	117 (18)	123 (17)
Diastolic BP (mmHg)	70 (12)	73 (14)
CRP (mg/L)	2.5 (3.7)	3.3 (4.8)

^*^Age, *n* = 125; BMI, waist circumference, 2 h glucose, systolic and diastolic blood pressure, *n* = 126; Lp(a), *n* = 67

^**^Age, LDL-c, HDL-C, *n* = 74; BMI and 2-h glucose, *n* = 75; Lp(a), *n* = 49

BMI, body mass index, HbA1c, glycated hemoglobin, C, cholesterol, LDL, low-density lipoprotein, HDL, high-density lipoprotein, Lp(a), lipoprotein a, BP, blood pressure, CRP, C-reactive protein

### Association between food intake and 2-h glucose in the whole sample

**Fig. 3:**
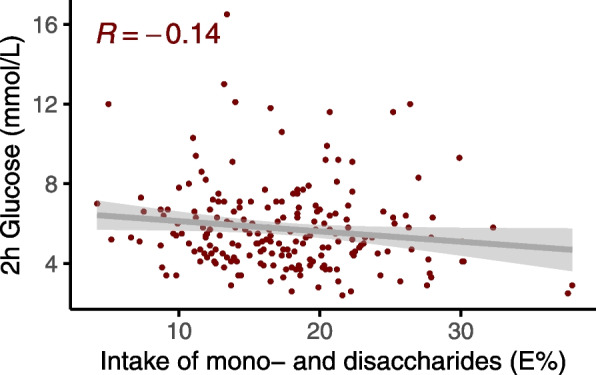
Association between intake of mono and disaccharides (energy %) and 2-h glucose. Intake of mono and disaccharides (energy %) was associated with 2-h glucose (*p* = 0.02) after an oral glucose tolerance test in the whole sample

We analyzed the association between intake of all food groups (Table 1) and 2-h glucose after an OGTT, adjusted for sex, age, BMI, use of statins, smoking, and energy intake. The associations between intake of macronutrients (E%) and 2-h glucose were analyzed with the same model, but not adjusted for energy intake. There were no significant association between intake of any food group and 2-h glucose (Supplemental file 2). However, higher intake of mono- and disaccharides (E%) were associated with a lower 2-h glucose (Fig. 3, *p* = 0.02). This association remained after adjusting for multiple comparisons (FDR < 10%). All food intake 2-h glucose associations in the whole population are visualized in Supplemental Fig. 3 and Supplemental Fig. 4.

**Fig. 4:**
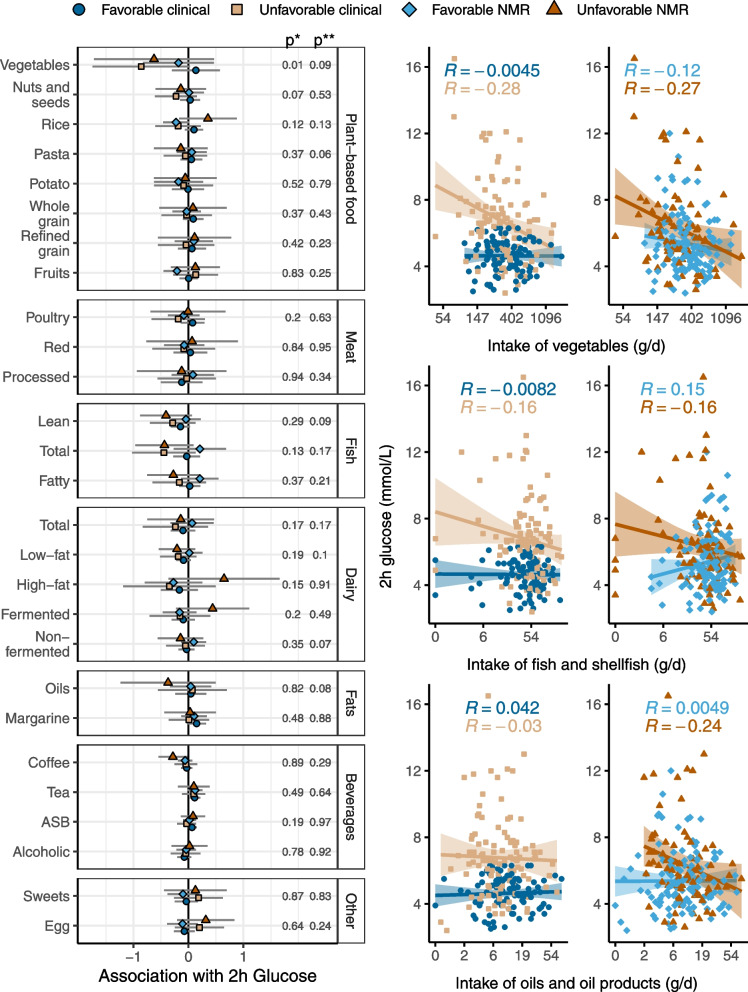
Interaction between intake of food groups and metabotypes on the association with 2-h glucose. The forest plot to the left shows β-coefficients for the association between intake of food groups and 2-h glucose for the different metabotypes, adjusted for sex, age, BMI, use of statins, smoking, and energy intake. p*, *p* value of the metabotype-food group interaction term for the clinical metabotypes. p**, *p* value of the metabotype-food group interaction term for the NMR metabotypes. The scatter plots show the unadjusted correlations between 2-h glucose and three highlighted food groups for the clinical metabotypes (to the left) and the NMR metabotypes (to the right)

### Interactions between food group intake and metabotypes on the association with 2-h glucose

We investigated if there were any interactions between the metabotypes and intake of food groups on the associations with 2-h glucose, adjusted for sex, age, BMI, statin use, smoking, and energy intake. All regression coefficients, 95% confidence intervals, *p* values, and FDR *q* values from these analyses can be found in Supplemental file 3. There was an interaction between the clinical metabotypes and intake of vegetables (Fig. 4, *p* = 0.01). Individuals with a low vegetable intake in the unfavorable clinical metabotype had higher postprandial blood glucose peaks compared to the favorable clinical metabotype. This suggests that the unfavorable clinical metabotype may improve their glucose tolerance by eating more vegetables. The same pattern was seen for the NMR metabotypes, but the separation of these metabotypes was less clear than for the clinical metabotypes. In the whole sample, the association between glucose tolerance and food intake depended on age for intake of fruit (*p* = 0.01) and intake of vegetables (*p* = 0.03, Supplemental file 4).

**Fig. 5:**
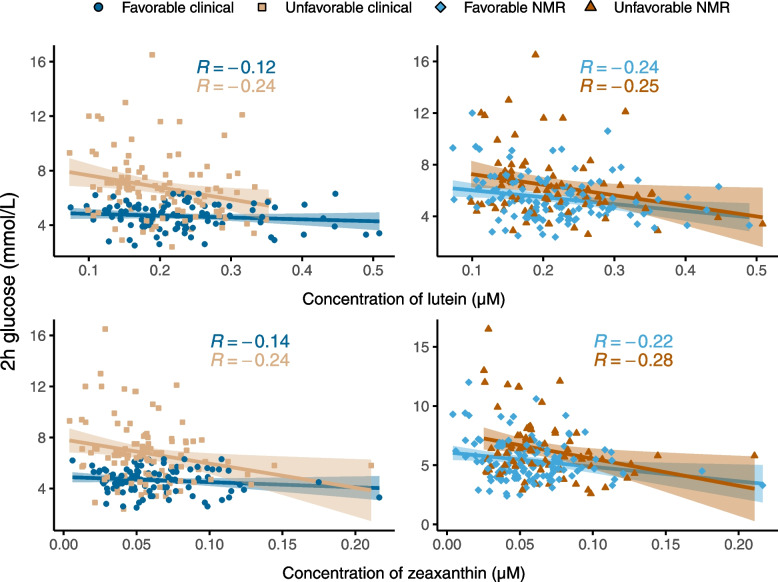
Unadjusted correlations between 2-h glucose and lutein (top) and zeaxanthin (bottom)

To verify the interaction between the clinical metabotypes and vegetable intake, we used objective biomarkers of vegetable intake and investigated if these interacted with the metabotypes on the association with 2-h glucose, adjusted for sex, age, BMI, statin use, smoking, and energy intake. We excluded one participant from the carotenoid analyses because of regular intake of several carotenoid containing dietary supplements. Of the six different carotenoids we analyzed in plasma, there was a significant interaction between the clinical metabotypes and plasma levels of lutein (*p* = 0.04) and zeaxanthin (*p* = 0.04). In addition, there was a significant interaction between the NMR metabotype and zeaxanthin (*p* = 0.02). Unadjusted correlation between the 2-h glucose and plasma lutein and zeaxanthin in the different metabotypes are shown in Fig. 5. All regression coefficients, 95% confidence intervals and *p* values from the carotenoid analyses can be found in Supplemental file 5.

There were no other significant food group—metabotype interactions; nonetheless, there seemed to be differences between the clinical and NMR metabotypes related to some of the food groups. Although not significantly, dietary fat sources, including fish and shellfish, high-fat and fermented dairy products, and vegetable oils and oil products, seemed to have a stronger association with 2-h glucose in the unfavorable NMR metabotype than the other metabotypes. For example, intake of fish and shellfish was associated with 2-h glucose in the opposite directions for the NMR metabotypes (interaction, *p* = 0.17). A high intake was associated with lower 2-h glucose in the unfavorable and higher 2-h glucose in the unfavorable NMR metabotype. The associations between fish intake and 2-h glucose were more similar for the clinical metabotypes. Finally, higher intake of oil and oil products was associated with lower 2-h glucose levels in the unfavorable NMR metabotype, but not in the favorable NMR metabotype (interaction, *p* = 0.08). Overall, the data suggest that higher intake of foods high in polyunsaturated fatty acids (PUFA) may be associated with lower 2-h blood glucose in the unfavorable NMR metabotype, while there is no such association in the favorable NMR metabotype. In the whole sample, the association between glucose tolerance and food intake depended on sex for intake of high-fat dairy (*p* = 0.01) and intake of *cis*-PUFA (*p* = 0.04, Supplemental file 4).

### Interaction between intake of macronutrients and metabotypes on the association with 2-h glucose

**Fig. 6:**
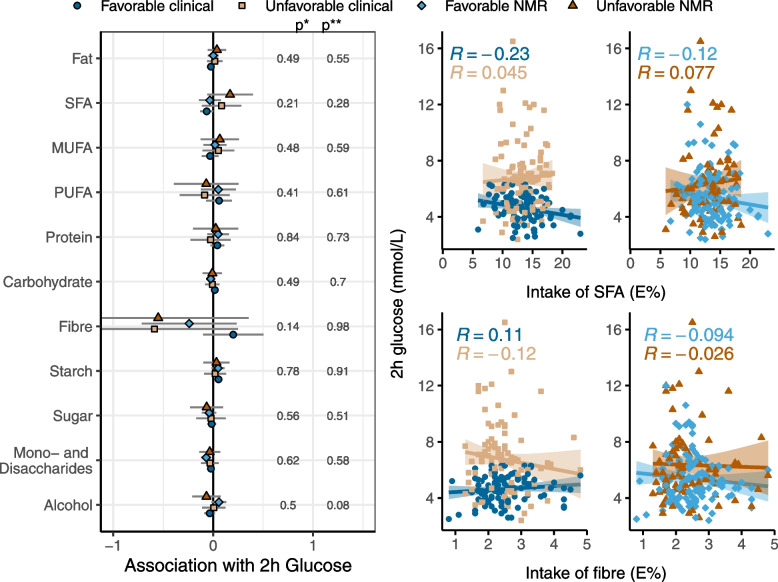
Interaction between intake of macronutrients and metabotypes on the association with 2-h glucose*.* The forest plot to the left shows β-coefficients for the association between intake of nutrients and 2-h glucose for the different metabotypes, adjusted for sex, age, BMI, use of statins, and smoking. p*, *p* value of the metabotype-nutrient interaction term for the clinical metabotypes. p**, *p* value of the metabotype-nutrient interaction term for the NMR metabotypes. The scatter plots show the unadjusted correlations between 2-h glucose and two highlighted nutrients for the clinical metabotypes (to the left) and the NMR metabotypes (to the right)

There were no significant interactions between intake of macronutrients and metabotypes on the association with 2-h glucose (Supplemental file 6). Although not significantly, a higher intake of fiber was associated with a lower 2-h glucose for the unfavorable clinical metabotype compared to the favorable clinical metabotype (interaction, *p* = 0.14, Fig. 6). Finally, a higher intake of SFA was non-significantly associated with higher 2-h glucose in the unfavorable NMR metabotype, while the SFA-2-h glucose association was less pronounced in the other metabotypes.

## Discussion

In this study, we grouped participants into metabotypes based on standard clinical cutoffs for glycemic variables and based on NMR metabolomics. For the clinical metabotypes, there was an interaction with intake of vegetables on the association with glucose tolerance. Although there were no other statistically significant interactions, the association between glucose tolerance and intake of fiber depended on the clinical metabotypes. Finally, the association between glucose tolerance and intake of saturated fatty acids and dietary fat sources, such as vegetable oils, depended non-significantly on the NMR metabotypes.

It seemed like the unfavorable metabotypes, regardless of how they were generated, would have a greater benefit on glucose tolerance of improving the diet than the favorable metabotypes. However, in all metabotypes, intake of food groups including nuts and seeds, lean fish, and low-fat dairy were associated with lower 2-h glucose (Fig. 4). This supports that advice to consume more of these food groups in food based dietary guidelines are beneficial, regardless of metabolic phenotype and disease risk. In contrast, intake of food groups such as vegetables and fish seemed to be more beneficial for the unfavorable than the favorable metabotypes. Future metabotype studies should investigate if a healthy diet is even more important to prevent lifestyle diseases in individuals with a deteriorated metabolic phenotype.

Higher intake of vegetables was associated with better glucose tolerance in the unfavorable clinical metabotype, while there was no such association in the favorable clinical metabotype. The same pattern was seen for intake of dietary fiber. There was a great difference in the average age between the favorable and the unfavorable clinical metabotype, 44 and 55 years, respectively. Moreover, the association between glucose tolerance and food intake depended on age for intake of fruit and vegetables. Hence, the age difference between the clinical metabotypes is important for the interaction between vegetable intake and the clinical metabotypes. Although we have adjusted for age in the food intake-metabotype interaction analyses, we cannot rule out residual confounding. A meta-analysis of prospective cohort studies that included participants free of T2DM at onset of the study showed a non-significant inverse association between intake of vegetables and risk of T2DM [5]. However, the range of vegetable intake in this meta-analysis was narrower than in our study. Similarly, a meta-analysis of prospective cohort studies showed that intake of dietary fiber was associated with a reduced T2DM risk only in certain geographic regions [24]. Our study suggest that the metabolic phenotype may modulate the association between vegetable intake and glucose tolerance. It is also possible that vegetable intake modulates the metabolic phenotype and thus the association with glucose tolerance. Hence, this interaction may explain conflicting results in studies examining associations between vegetable and fiber intake and T2DM risk [5]. Moreover, an 8-week whole grain diet intervention improved glucose tolerance in obese adults compared to a refined grain diet [25]. Hence, the differences between metabotypes in associations between glucose tolerance and vegetable and fiber intake are probably driven by the separation of the clinical metabotypes by glycemic variables.

The interaction between the clinical metabotypes and vegetable intake was confirmed by analyses of plasma levels of carotenoids, objective biomarkers of vegetable intake [26]. High plasma levels of both lutein and zeaxanthin were associated with better glucose tolerance in the unfavorable clinical metabotype, while there was no such association in the favorable clinical metabotype. The plasma level of zeaxanthin also had a significant interaction with the NMR metabotypes. This may reflect that there were similar patterns for the association between vegetable intake and glucose tolerance in the NMR metabotypes and the clinical metabotypes, although the interaction was not significant for the NMR metabotypes.

The association between food intake and glucose tolerance depended on the NMR metabotypes for several dietary fat sources and SFA, although these interactions were non-significant. A high intake of dietary sources of PUFA such as fish and vegetable oils and oil products were associated with improved glucose tolerance in the unfavorable NMR metabotype, but not in the favorable. Correspondingly, a high intake of SFA as well as dietary sources of SFA such as high-fat and fermented dairy products were non-significantly associated with a worsened glucose tolerance in the unfavorable NMR metabotype, but not the favorable. The distribution of male and female participants differed between the NMR metabotypes, and the association between glucose tolerance and food intake depended on sex for high-fat dairy and *cis*-PUFA. Although we adjusted for sex in the food intake-metabotype interaction analyses, and although the NMR metabotypes were very similar after removing variation associated with sex, there may still be residual confounding by sex. Differences in the concentration of lipoprotein particles and their lipid content were the main drivers of the separation of the favorable and unfavorable NMR metabotypes. This suggests that an improved dietary fat quality that would lower LDL-C also would improve glucose tolerance in the unfavorable NMR metabotype. One possible explanation would be that in people with elevated LDL-C, pancreatic β cells accumulate cholesterol due to uptake via the LDL receptor which is abundantly expressed in pancreatic β cells [27]. Accumulation of cholesterol in β cells causes a reduction of the cells’ glucose stimulated insulin secretion and prolonged exposure to LDL-C may lead to beta-cell death [28–30]. Hence, lowering of LDL-C will improve glucose tolerance. A sufficiently powered study is needed to confirm the non-significant interactions between metabotypes separated mainly by LDL-C and dietary fat sources with glucose tolerance.

Surprisingly, higher intake of mono- and disaccharides was associated with improved glucose tolerance in the whole study population. This may be because people with an unhealthy lifestyle tend to underreport intake of unhealthy foods, such as foods with a high content of simple sugars [31, 32]. However, this finding may also be spurious, as we did not find any other associations between intake of other groups of unhealthy foods and glucose tolerance in the whole study population.

In this study, we chose to split our study population into only two different metabotypes per strategy because splitting the data into even smaller groups would results in analyses with too low power. In addition, the two metabotypes generated per strategy differed in clinically relevant variables that made it possible to classify the metabotypes as favorable and unfavorable. However, a larger study population would have enabled the generation of more metabotypes, allowing comparison of more clearly separated groups that would have affected the analyses of metabotype-food intake interactions.

There is no consensus on how to define metabotypes; thus, the term “metabotype” is subjectively used and metabotypes are constructed to fit the aims of the individual studies [6]. Many studies have used a handful of selected metabolites related to the metabolic syndrome and cardiovascular disease to create metabotypes [6]. As an example, metabotypes based on the glucose response following an OGTT differed in BMI, body fat, triglycerides, hsCRP, insulin response, and β-cell function [33]. Moreover, clustering of participants using triglycerides, total cholesterol, HDL-cholesterol, and glucose identified three metabotypes that were given targeted dietary advice based on the biochemical characteristics of each cluster. The targeted advice largely agreed with personalized dietary advice based on individual characteristics, demonstrating that metabotypes are useful in precision nutrition [34]. Metabotypes based on a few selected variables as well as metabotypes based on omics-technologies have been used to study the relationship between metabotype and food intake on disease risk. In a metabotype characterized by a high proportion of T2DM, as well as high age, BMI and waist circumference, a low intake of fruit and a high intake of sugar-sweetened beverages was associated with T2DM. In the same study, the more healthy metabotype showed associations between meat intake and T2DM, demonstrating that different metabotypes may benefit from different dietary advice to achieve disease risk reduction [7]. Furthermore, NMR metabolomics has been used to determine metabotypes that responded to vitamin D supplementation by improving metabolic syndrome-related risk markers including CRP, insulin, and HOMA scores [35]. Finally, lipoprotein profiles from NMR metabolomics were used to study the response to fenofibrate in clusters of low, medium, and high degree of dyslipidemia. This clustering approach was better at separating those with a beneficial response to fenofibrate therapy than standard clinical methods [36].

The NMR platform that was used in this study quantifies 250 metabolites; however, the vast majority of these variables are related to lipoproteins, lipids, and fatty acids. Hence, the metabotypes based on NMR data are distinguished by a favorable and unfavorable lipid profile. The use of other untargeted and targeted metabolomics platforms covering different aspects of metabolism would have produced metabotypes characterized on other metabolites than lipids. Moreover, there were more men in the unfavorable NMR metabotype compared to the favorable metabotype. This could potentially have been a driver of both the differences in NMR metabotype characteristics, and the food intake associations. However, the variation in the NMR data introduced by sex was removed when the residuals were clustered, and the clustering of residuals approach generated very similar metabotypes as the two other clustering approaches.

It is possible that the use of LDL-C, in addition to other standard lipid variables, would generate metabotypes similar to the NMR metabotypes generated in this study. Hence, the use of NMR metabolomics may not provide us with more useful metabotypes compared to standard clinical variables. If there is a clinically relevant difference between the metabotypes, e.g., a difference in fasting glucose or LDL-C, it is not surprising that this difference is the main driver of the associations compared to fluctuations in a wider range of molecules with a less important role in determining disease risk. In other words, more research is needed to determine if the cost of creating metabotypes based on omics-technologies can be justified.

This exploratory study is limited by a low sample size that increases the probability of both positive and negative findings being due to chance. Furthermore, although we adjusted for sex and age in the analyses of interaction between metabotypes and food intake on glucose tolerance, there may still be residual confounding by sex and age. Specifically, sex and age differences between the metabotypes may contribute to bias related to body composition, dietary habits, and drug use. Moreover, we investigated interactions between food intake and 2-h glucose in an OGTT, while measuring glucose at more time points and analyzing the whole glucose response curve may have provided a better estimation of glucose tolerance. Finally, this study is observational and cannot infer causality, especially due to residual confounding and the possibility of reverse causality. The study is strengthened by robust metabotypes that remained very similar with the different clustering approaches that were applied, although we cannot exclude that this may be due to overfitting of the models. Furthermore, this study demonstrates that it is possible to generate metabotypes based on both a simple set and a more complex set of variables. However, it is not clear if the cost of doing omics-analyses in metabotyping can be justified by producing more informative metabotypes compared to metabotypes based on a few selected variables with a strong disease risk association. Finally, the association between food intake and glucose tolerance were dependent of metabotype, suggesting that a similar approach can be used to guide the design of metabotype-specific interventions in future studies in precision nutrition.

## Conclusions

The metabotypes with more unfavorable characteristics showed stronger associations between food intake and glucose tolerance than the more favorable metabotypes. Moreover, the variables used to create the metabotypes affected how the metabotypes interacted with dietary intake on the association with glucose tolerance. Metabotyping may be a useful tool in precision nutrition to find dietary interventions that will benefit specific groups of individuals.

## Supplementary Information

**Additional file 1: Supplemental Figure 1.**Variance explained by the first ten principal components. PC, principal component File format: .pdf

**Additional file 2: Supplemental Figure 2. **Separation of participants into metabotypes. A) Separation of the favorable and the unfavorable clinical metabotype based on fasting glucose (cut-off = 5.6 mmol/L), 2h OGTT glucose (cut-off = 6.5 mmol/L) and HbA1c (cut-off = 5.8 %). B) Separation of the favorable and unfavorable NMR metabotype generated by k-means clustering of scaled NMR metabolomics data directly, visualized by the first four PCs. C) Separation of the favorable and unfavorable NMR metabotype generated by k-means clustering of the first four PCs, visualized by the four PCs. D) Separation of the favorable and unfavorable NMR metabotype generated by k-means clustering of residuals from regression models of the NMR variables adjusted for sex, age, BMI, statin use and smoking status, visualized by the first four PCs. Note the similarity between panels B and C, as expected. OGTT, oral glucose tolerance test, PC, principal component. File format: .pdf

**Additional file 3: Supplemental Figure 3. **Associations between intake of food groups and 2h glucose in the whole population. β-coefficients, with 95% confidence intervals, for the association between intake of food groups and 2h glucose in the whole population, adjusted for sex, age, BMI, use of statins, smoking and energy intake. Numbers to the right are *p*-values of the association. ASB, artificially sweetened beverages. File format: .pdf

**Additional file 4: Supplemental Figure 4.** Associations between intake of macronutrients and 2h glucose in the whole population. β-coefficients, with 95% confidence intervals, for the association between intake of macronutrients (E%) and 2h glucose in the whole population, adjusted for sex, age, BMI, use of statins and smoking. Numbers to the right are p-values of the association. MUFA, monounsaturated fatty acids, PUFA, polyunsaturated fatty acids, SFA, saturated fatty acids File format: .pdf

**Additional file 5: Supplemental file 1.** List of NMR variables used in clustering analyses. File format: .xlsx

**Additional file 6: Supplemental file 2.** Association between 2h glucose and intake of food groups and nutrients in all participants. Regression coefficients, 95% confidence intervals, p-values and FDR q-values for regression analyses. Association between 2h glucose and intake of food groups, adjusted for sex, age, BMI, statin use, smoking and energy intake. Association between 2h glucose and intake of nutrients, adjusted for sex, age, BMI, statin use and smoking. File format: .xlsx

**Additional file 7: Supplemental file 3.** Interactions between metabotypes and intake of food groups on the associations with 2h glucose. Regression coefficients, 95% confidence intervals, p-values for regression analyses. FDR q-values for the metabotype-food group interaction effect. Model adjusted for sex, age, BMI, statin use, smoking and energy intake File format: .xlsx

**Additional file 8: Supplemental file 4.** Interaction between age and food intake on the association with 2h glucose and interaction between sex and food intake on the association with 2h glucose. Regression coefficients and p-values for regression analyses. File format: .xlsx

**Additional file 9: Supplemental file 5.** Interactions between metabotypes and carotenoid concentrations on the associations with 2h glucose. Regression coefficients, 95% confidence intervals, *p*-values for regression analyses. Model adjusted for sex, age, BMI, statin use, smoking and energy intake. File format: .xlsx

**Additional file 10: Supplemental file 6.** Interactions between metabotypes and nutrient intake on the associations with 2h glucose. Regression coefficients, 95% confidence intervals, p-values for regression analyses. FDR q-values for the metabotype-nutrient interaction effect. Model adjusted for sex, age, BMI, statin use and smoking. File format: .xlsx

## Data Availability

The datasets used and analyzed during the current study are available from the corresponding author upon reasonable request.

## Abbreviations

BMI: Body mass index
FFQ: Food frequency questionnaire
HDL-C: High-density lipoprotein cholesterol
LDL-C: Low-density lipoprotein cholesterol
NMR: Nuclear magnetic resonance
OGTT: Oral glucose tolerance test
PC: Principal component
PCA: Principal component analysis
SFA: Saturated fatty acids
T2DM: Type 2 diabetes mellitus
VLDL: Very low-density lipoprotein
