# Supplementary figures and images for: Associations between dietary intake and glucose tolerance in clinical and metabolomics-based metabotypes
Source: Genes Nutr. 2023 Mar 10;18:3. doi: 10.1186/s12263-023-00721-6 (PMC10007735; doi:10.1186/s12263-023-00721-6)

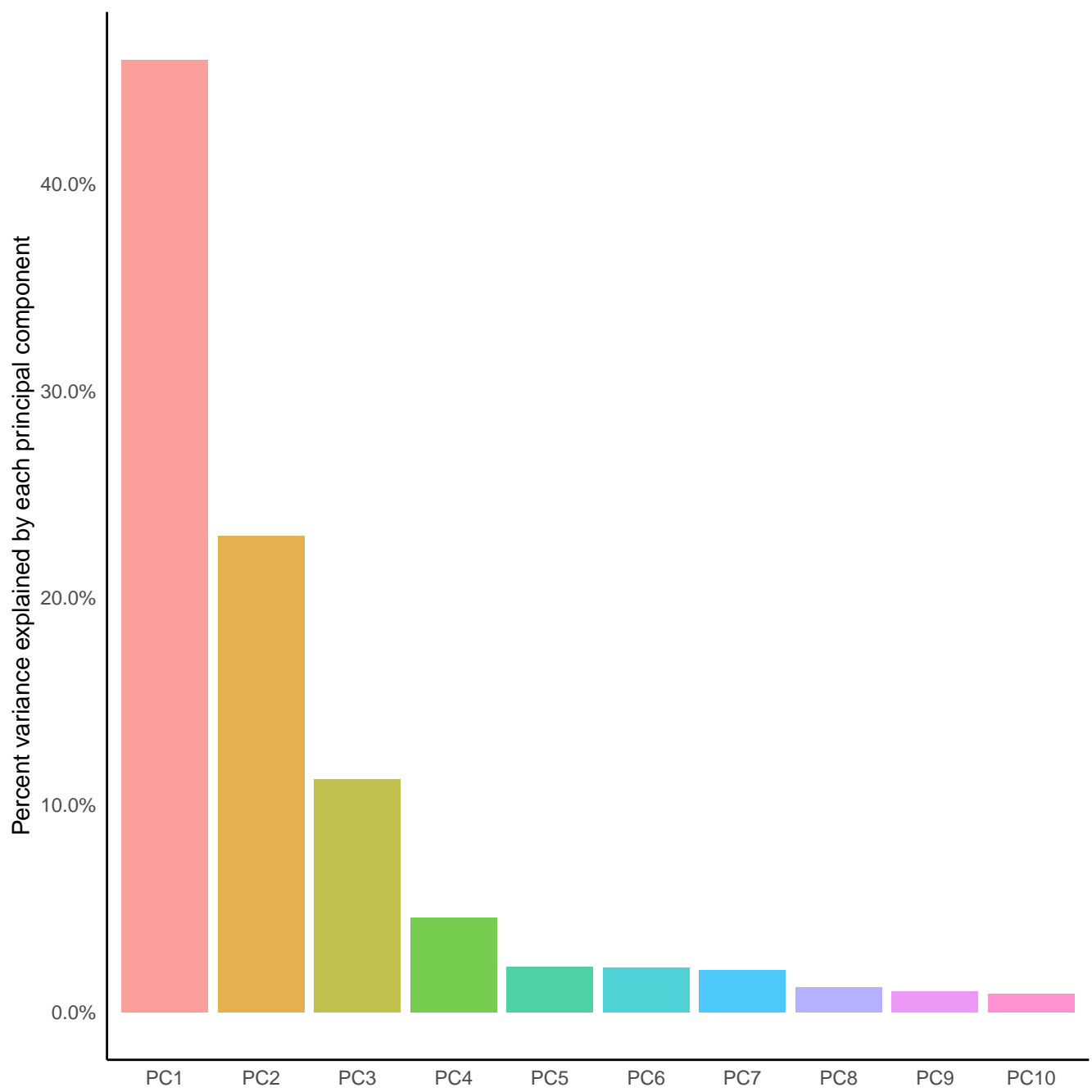

Supplement: Supplementary file 1 — Additional file 1: Supplemental Figure 1.Variance explained by the first ten principal components. PC, principal component File format: .pdf [file 12263_2023_721_MOESM1_ESM.pdf]

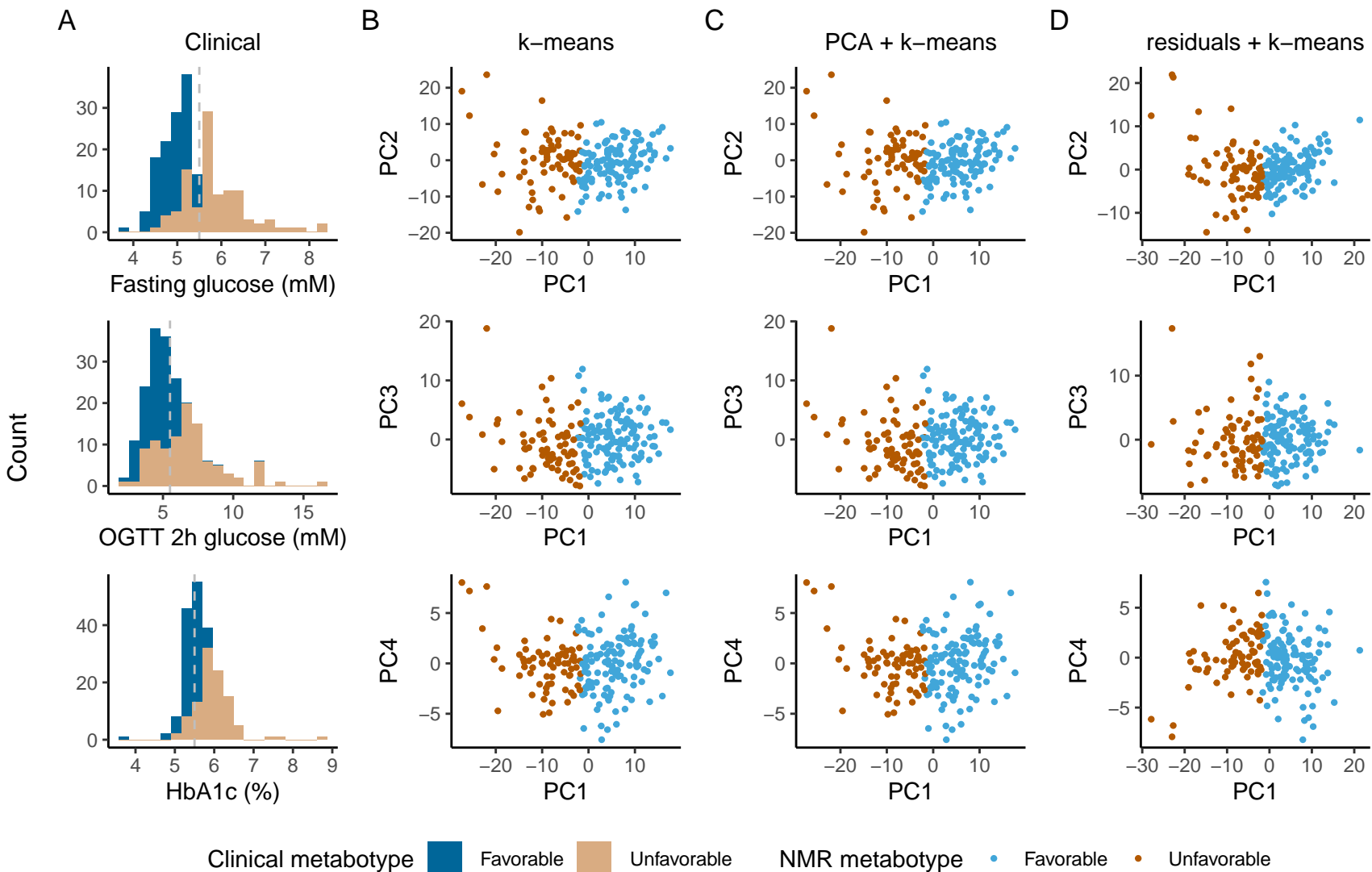

Supplement: Supplementary file 2 — Additional file 2: Supplemental Figure 2. Separation of participants into metabotypes. A) Separation of the favorable and the unfavorable clinical metabotype based on fasting glucose (cut-off = 5.6 mmol/L), 2h OGTT glucose (cut-off = 6.5 mmol/L) and HbA1c (cut-off = 5.8 %). B) Separation of the favorable and unfavorable NMR metabotype generated by k-means clustering of scaled NMR metabolomics data directly, visualized by the first four PCs. C) Separation of the favorable and unfavorable NMR metabotype generated by k-means clustering of the first four PCs, visualized by the four PCs. D) Separation of the favorable and unfavorable NMR metabotype generated by k-means clustering of residuals from regression models of the NMR variables adjusted for sex, age, BMI, statin use and smoking status, visualized by the first four PCs. Note the similarity between panels B and C, as expected. OGTT, oral glucose tolerance test, PC, principal component. File format: .pdf [file 12263_2023_721_MOESM2_ESM.pdf]

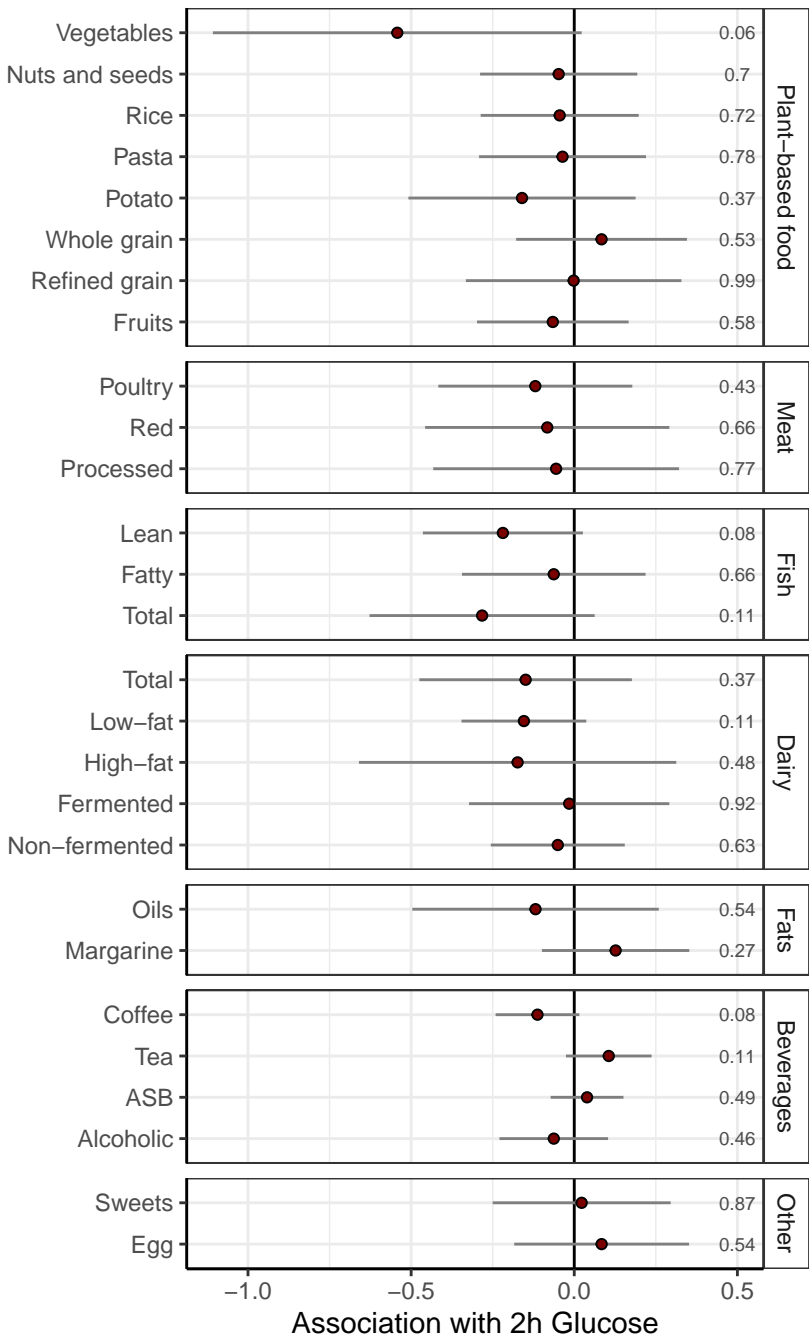

Supplement: Supplementary file 3 — Additional file 3: Supplemental Figure 3. Associations between intake of food groups and 2h glucose in the whole population. β-coefficients, with 95% confidence intervals, for the association between intake of food groups and 2h glucose in the whole population, adjusted for sex, age, BMI, use of statins, smoking and energy intake. Numbers to the right are p-values of the association. ASB, artificially sweetened beverages. File format: .pdf [file 12263_2023_721_MOESM3_ESM.pdf]

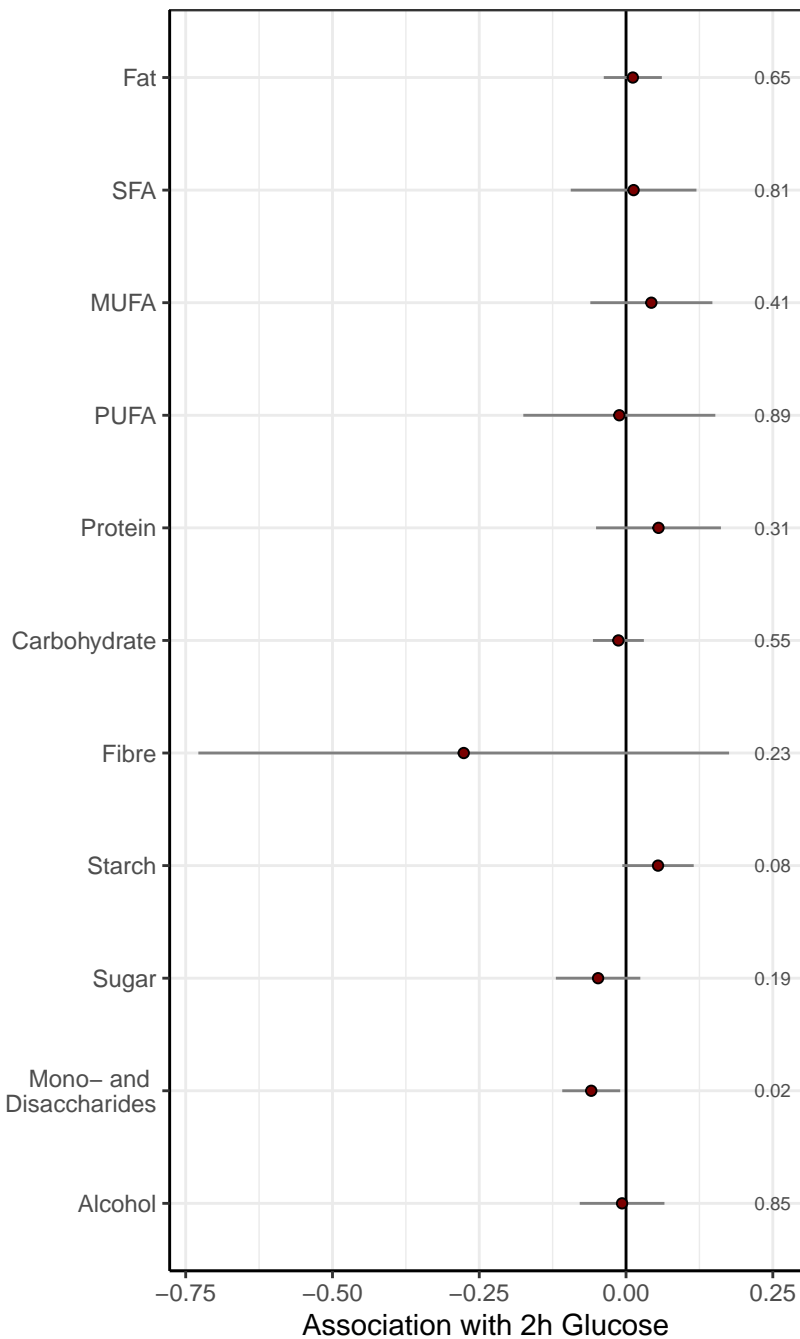

Supplement: Supplementary file 4 — Additional file 4: Supplemental Figure 4. Associations between intake of macronutrients and 2h glucose in the whole population. β-coefficients, with 95% confidence intervals, for the association between intake of macronutrients (E%) and 2h glucose in the whole population, adjusted for sex, age, BMI, use of statins and smoking. Numbers to the right are p-values of the association. MUFA, monounsaturated fatty acids, PUFA, polyunsaturated fatty acids, SFA, saturated fatty acids File format: .pdf [file 12263_2023_721_MOESM4_ESM.pdf]
